# An Update on the Lived Experience of Dry Mouth in Sjögren's Syndrome Patients

**DOI:** 10.3389/froh.2021.767568

**Published:** 2021-11-02

**Authors:** Di Ying Joanna Ngo, William Murray Thomson

**Affiliations:** ^1^Auckland District Health Board, Auckland, New Zealand; ^2^Department of Oral Sciences, University of Otago Dental School, Dunedin, New Zealand

**Keywords:** Sjögren's Syndrome, dry mouth impact, salivary gland hypofunction, xerostomia, qualitative, lived experience

## Abstract

This paper aims to provide an update on research findings on the lived experience of dry mouth in Sjögren's Syndrome (SS) patients. Dry mouth is a significant condition that impacts on the daily lives of people with SS. There will first be a summary of the definition, etiology, and manifestation of dry mouth in SS patients. There will next be an overview of the measurement of the impact of dry mouth on the quality of life in SS patients. This will include a deliberation of both quantitative and qualitative methods. Lastly, there will be discussion on the consequences of dry mouth, with a focus on qualitative studies that seek to understand patients' physical, emotional, and social domains of life.

## Introduction

Sjögren's Syndrome (SS) is a chronic autoimmune disorder that is distinguished by the lymphocytic infiltration of exocrine glands in various sites [1]. Primary SS manifests as dry mouth and dry eyes. In SS associated with other autoimmune diseases, dry eyes and dry mouth occur together with other autoimmune disorders such as rheumatoid arthritis or systemic lupus erythematosus [2]. In the American-European Consensus Group (AECG) Revised International Classification criteria for SS, dry mouth is one of the main classification criteria for the diagnosis of Sjögren's Syndrome (SS) [3]. More recently, the American College of Rheumatology (ACR) classification includes only the histopathology of salivary gland biopsy as a criterion [4]. Nonetheless, both sets of classification yield concordant results in the diagnosis of SS. The current 2016 ACR/European League Against Rheumatism (EULAR) classification criteria for primary SS include the objective measurement of dry mouth (unstimulated whole saliva flow rate) that can be applied to a patient with a subjective symptom of dry mouth [5].

SS is estimated to affect 0.5–1.0% of the population and occurs more commonly in middle-aged women [6]. Dry mouth has a subjective component of xerostomia, and an objective component of salivary gland hypofunction (SGH) [7, 8]. In the Sjögren's International Collaborative Clinical Alliance (SICCA) registry of 1578 SS participants, 90% reported symptoms of dry mouth and 62% were measured to have salivary gland hypofunction [9]. Health related quality of life (HRQoL) indices have been used to measure the negative affect of dry mouth on the quality of life of SS patients [10–13]. Some of these multi-item measures include the 36-Item Short-Form Health Survey {SF-36; [14]}, the Oral Health Impact Profile {OHIP [15]}, and the Geriatric/General Oral Health Assessment Index {GOHAI; [16]}. There are limitations to the use of such indices in the capturing of the extent of the impact of SS-associated dry mouth on domains of daily life [17]. Qualitative studies [10–13] seek to understand the effect of dry mouth on SS patients' physical, emotional, and social domains of life. This paper aims to provide a review and update on the lived experiences of dry mouth in SS patients.

## Definition, Etiology, and Manifestation of Dry Mouth in SS Patients

Dry mouth can manifest as xerostomia or SGH [7, 8]. Xerostomia is the subjective symptom of dry mouth and is evaluated by questioning the individual [18]. SGH is the objective decrease in saliva production and is measured by sialometry [19]. The relationship between xerostomia and SGH is complex and both conditions can occur independently of each other [20]. In an American study of 169 SS patients, 93.5% reported dry mouth, 55.6% reported a sore mouth, 41.4% reported dysgeusia, 25.4% reported difficulty in swallowing, and 16.6% reported difficulty in chewing [21]. A more recent Chinese study of 224 primary SS patients, reported dry mouth in 96.0% and this was predicted by age, fatigue, and total pain [22].

The pathogenesis of dry mouth in SS is complex. It has been hypothesized to involve a series of inflammatory changes that result in damage or inhibition of the salivary glands and resulting decrease in saliva production. These include: (1) a disruption of epithelial cells; (2) T lymphocytes migration to the glands; (3) previously thought glandular damage triggered by SS (ANA, anti-Ro/SSA, anti-LA/SSB, and rheumatoid factor) autoantibodies in advanced disease [23] or potentially by novel autoantibodies anti-salivary gland protein 1 (SP1), anti-carbonic anhydrase 6 (CA6), and parotid secretory protein (PSP) in early disease [24, 25]; (4) more recent theory of autoantibodies mediated inhibition of type 3 muscarinic cholinergic receptor [26]; (5) activation of the cytokines and interferon pathways; and (6) structural destruction of acinar cells [27]. It has also been suggested that the quality of saliva is altered by changes in secretory route and salivary mucins [28].

Saliva plays an essential role in numerous functions of the mouth, such as protecting the oral cavity (lubrication, buffering, and antibacterial activity), taste, digestion, and speech [29]. The impact of dry mouth on the SS patient is because of the compromise of these salivary functions.

Dry mouth in SS patients can manifest as: (1) dry, cracked, and peeling lips; (2) a dry and coarse tongue; (3) cracks in the corner of the mouth; (4) an erythematous tongue; and (5) oral ulcerations [30]. The lack of and altered saliva quality results in difficulty in swallowing, speech problems, severe and progressive dental caries, dental erosion, salivary gland swelling, mucositis, or oral candidiasis [31]. There is no cure for SS (and the associated dry mouth), and its management involves dealing with the underlying systemic conditions(s), alleviating symptoms, and instituting measures to prevent secondary complications [32, 33].

## Measurement of the Impact of Dry Mouth in the Quality of Life in SS Patients

HRQoL and oral-health-related quality of life (OHRQoL) consider the physical, psychological, and social aspects of general and oral health. HRQoL considers different aspects of health, such as function and well-being [34]. OHRQoL is a conceptual framework that refers to the extent to which oral disorders disrupt an individual's day-to-day function [35]. Both HRQoL and OHRQoL are subjective and can be measured using single-item methods or multi-item summated rating scales (the latter captures a more complete profile, while the former provide a global assessment). The SF-36 is the most commonly used multidimensional measure for HRQoL. The OHIP-14 is a widely used OHRQoL measure, while the GOHAI is popular for assessing OHRQoL in older people. The SF-36 was designed to measure different dimensions of HRQoL, including: (1) physical functioning; (2) social functioning; (3) physical role limitations (4) emotional role limitations; (5) mental health; (6) energy; (7) pain; and (8) general health perceptions. It is easy to use, acceptable to patients, and is reliable and valid [36]. Similarly the OHIP-14 gathers information in seven domains including: (1) functional limitation; (2) pain; (3) physical disability; (4) psychological discomfort; (5) psychological disability; (6) social disability; and (7) handicap. It has demonstrated good reliability, validity, and responsiveness in the general population [37].

A United States study of 547 primary SS sufferers used the SF-36 to demonstrate that they had poor HRQoL because of the functional impact of SS from symptoms which included dry mouth-related morbidity [38]. Similarly, a Chinese study used the SF-36 to compare 185 primary SS patients with 168 healthy controls; it found oral disorders and impaired swallowing to be the main predictors of poorer HRQoL [39]. In a Dutch study comparing 235 SS patients to age- and sex-matched controls from the general population, the former group scored lower on the SF-36. Having to use artificial saliva to alleviate dry mouth symptoms predicted poorer HRQoL in the SS patients [40]. A Norwegian study of 177 primary SS patients used both the SF-36 and OHIP-14. It found oral distress to be severe, prevalent and significantly greater in the sample than in age- and sex-matched normative data, with marked effects on the HRQoL [12]. Using the same scales, a smaller American study found lower salivary flow rate to correlate with poorer OHIP-14 scores, and oral health to independently have a negative influence on quality of life [41]. A more recent study found the mean OHIP-14 score to be significantly higher in primary SS patients and positively associated with dysgeusia, halitosis, and burning sensations in the tongue [42]. The GOHAI has been used to study the effectiveness of treatment modalities for dry mouth in SS patients [43].

It is clear from these findings that dry mouth (and its associated symptoms) can be detrimental to the physical, emotional, and social domains of SS patients' lives, in a complex and multi-faceted manner which is detectable at the group level. Moreover, the psychological distress has been demonstrated by higher levels of clinical anxiety (32%) and depression (48%) that has been reported in SS patients [44] to be associated with symptoms (including dry mouth) [45, 46]. It is therefore important to understand the effects of dry mouth on individuals.

Conceptual models of HRQoL and OHRQoL have been developed to link the multiple disease-affected domains of patients' lives in an attempt to make sense of them. The Ferrans et al. model (2005) [47] is a conceptual model that has been applied to understand the impact of disease on patients, improving the understanding of the multidimensional components and pathways of HRQoL and OHRQoL. However, it is not possible to use such scales to explore the extent and complexity of the impact of dry mouth on individuals, and there are limitations as to how such models can provide insight into patient perceptions in terms of difficulty in: (1) quantifying experience; (2) conceptualizing disease experience affected by individual and environmental characteristics; (3) conceptualizing changes in patient experience over time; and (4) conceptualizing the effect of treatment. Qualitative research has the advantage of the ability to generate deep insight into complex phenomena such as thoughts, beliefs, attitudes and behaviors [48]. The common research methods include interviews, focus groups, and direct observation. The analysis of qualitative data involves a number of alternative approaches. These include: (1) thematic analysis; (2) grounded theory; and (3) the framework approach. All seek to define the underlying analytical categories which describe and explain social phenomena [48].

For example, a Chilean study interviewed (semi-structured) 12 primary SS women and analyzed the data based on the principles of grounded theory and found dry mouth to have a profound influence on daily life. The severity of the consequences depended on individual experiences with the illness, social influences (social support and physical surroundings), and the psychological responses of the patient (adjustment and maladjustment). A reflection of individual illness experience was reflected in one of the responses:

ML: “*I would give all the gold in the world just to have a little more moisture (in my mouth).”*

**Figure 1:**
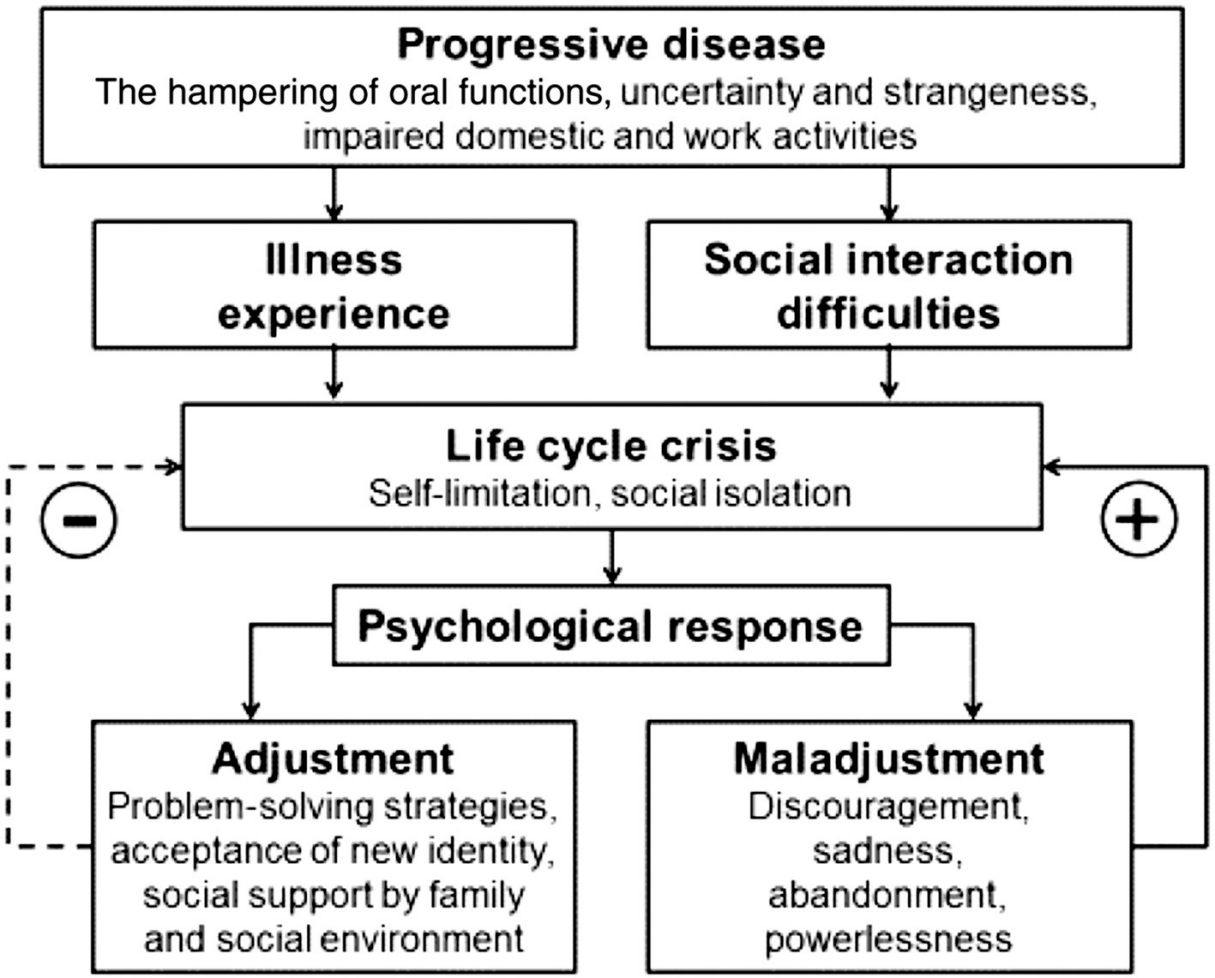
The integrated model summarizing the life experiences of women with primary SS and health-related behaviors [11].

The integrated model to summarize the life experiences of these women with primary SS is depicted in Figure 1. It was recommended that healthcare professionals should listen to SS sufferers and explore solutions based on a psychological approach [11].

A New Zealand qualitative study of 10 SS patients used diary entries and semi-structured interviews to explore how dry mouth affects their lives. Thematic content analysis was applied. The four main themes that depicted the lived experience of SS were: (1) the journey to diagnosis; (2) disease impact spectrum (of dry mouth amid other symptoms); (3) interactions with healthcare professionals; and (4) the positive coping process. The less-tangible impacts of dry mouth on quality of life were described during the interviews by the participants.

Amanda: “*It brings a lot of pain at times when you try and eat, clean your teeth and deal with the pain around my mouth when my skins flare up and my lips get so dry, they are constantly cracking. It is a constant battle trying to get everything lubricated. My water bottle is my best friend*.”

**Figure 2:**
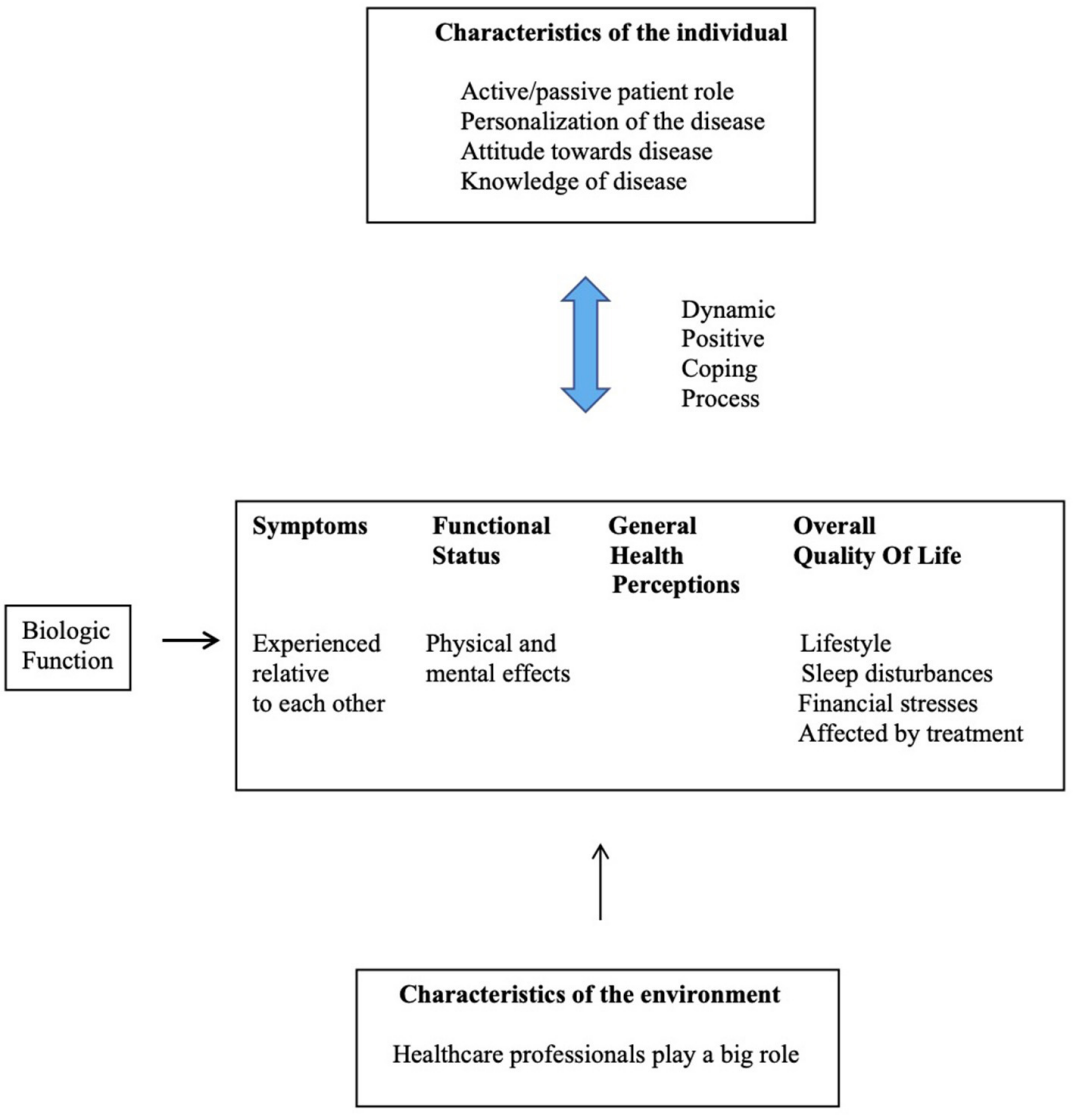
Adapted from [10]: The participants' view of the model of Ferrans et al. [47].

The participants' views were summarized with reference to the model depicted in Figure 2.

The study revealed that dry mouth was not a trivial symptom for SS sufferers, and that it has considerable impact on their day-to-day lives. An important message from this study was that healthcare professionals need to understand patients in the context of their individual characteristics and their respective environments [10].

A more recent qualitative study (from the United Kingdom) used semi-structured interviews with 17 SS patients to capture a comprehensive range of impacts of dry mouth on physical, emotional, and social functioning. The restriction on social participation as a result of dry mouth was described:

Jackie: “*I do see it as a problem when I go out for a meal somewhere and I look at it and I think… “Oh Gosh!! what can I actually choose of this menu?” and that is difficult…when you go out somewhere…”*

**Figure 3:**
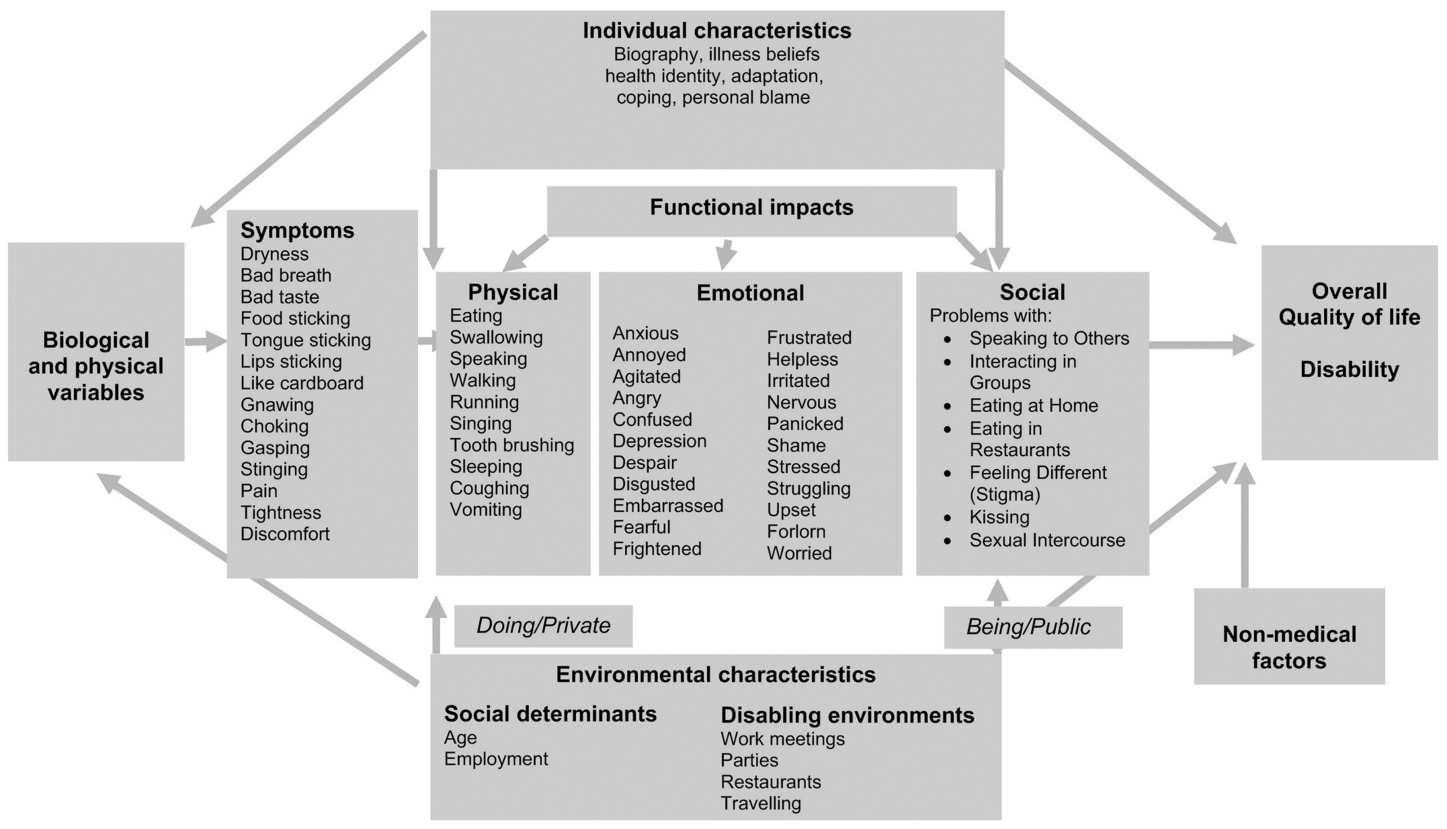
Adapted from [17]: The interaction between personal and environmental characteristics and the impacts of dry mouth in association with the Wilson and Cleary model [49].

The interaction between personal and environmental characteristics and the impact of dry mouth on the participants was summarized with reference to the Wilson and Cleary model [49], as depicted in Figure 3.

The recommendation from the study was to enhance the current measures of the impact of dry mouth in a comprehensive, systematic manner with reference to existing conceptual health models in order to focus on dry mouth interventions.

## Drawing it Together: SS Patients' Overall Lived Experience of Dry Mouth

The quantitative studies using quality of life indices demonstrate the range of impacts that dry mouth has on SS sufferers. The physical lack of saliva was found to be related to poorer OHIP-14 scores and oral health impacted on the general health and social function [41]. Moreover, the functional impact of dry mouth was determined to be specific to poor swallowing [39], dysgeusia, halitosis, and burning sensations in the tongue [42]. The other impacts on quality of life were a result of the management of dry mouth using saliva substitutes [40] and general oral distress [9]. Quantitative studies provide insight on the breadth of the effects that dry mouth can have on the daily lives of SS patients; supporting the biopsychosocial model [50] that health is not simply the lack of disease. However, no indices can comprehensively cover the full range of impacts of dry mouth on SS patients, nor can they explain how these affect each individual.

Qualitative studies have used conceptual health models in order to allow depth to the understanding of the impact of dry mouth and its meaning to individual SS sufferers. This is in the context of each SS patient's life, who they are, what their beliefs are, and the environment (including society and culture) in which they interact in. Dry mouth in SS patients has been found to be life-changing. The severity of the impact depended on individual illness experience, which can be affected by social support, which in turn, influences the psychological responses. The adjustment or maladjustment to SS as a progressive disease will then determine whether the individual experiences more social impacts such as social isolation and self-limitation [11]. This finding is consistent with and builds upon how primary SS patients reported poorer social functioning due to physical and emotional difficulties in a study using the SF-36 [41]. Moreover, the dry mouth symptoms in SS patients have been found to co-occur and be intertwined with other SS symptoms (such as fatigue and joint pain); thus, they comprise part of the mosaic of physical, psychological and social impacts. The effects of the symptoms on each SS patient were mapped to be understood in the context of their individual attitudes and dynamic coping mechanisms [10]. This enables improved understanding of how dry mouth is experienced amidst other SS symptoms (such as fatigue, tendomyalgia, articular involvement, and comorbidity) that, together with dry mouth, were found to be predictors of poor HRQoL determined with the SF-36 [40].

It was also highlighted that healthcare workers play a role in the environmental effects on disease impact [10]. The detailed impacts of dry mouth on SS patients' daily lives were captured in a systematic manner [17] according to the Wilson and Cleary conceptual model [49]. The strength of this particular study were in the wide range of domains covered in a systematic manner by mapping them to a well-established health model. There was also a comprehensive list of symptoms and their various functional impacts (physical, emotional, and social) that, in turn, determined the effects on overall quality of life and disability. This study introduced new approaches to consider dry mouth in a private and public setting. There was the concept of disabling environments; these included work meetings, parties, restaurants, and those encountered while traveling. This puts into context the social settings in which dry mouth produces disability in SS patients.

By contrast, quantitative studies can be useful for involving a large sample of patients to determine how dry mouth affects the different domains of quality of life in SS patients. The SF-36 and OHIP-14 indices are easy to use and allow comparison across different study samples. Qualitative studies can be realistically undertaken only with smaller samples, but they provide depth and contextualize how each individual is affected on a daily basis in different settings, depending on dynamic attitudes and disease stages. The different themes and conceptual models used build on the overall understanding of the impact of dry mouth on the daily lives of SS patients. However, qualitative interviews can be subjective and may be prone to interviewer bias [48]. Together, these two research approaches are synergistic and allow understanding of how the impacts recorded by quality of life indices affect individual patients and help inform the approaches that can be taken by healthcare workers. Future research directions should include mixed-methods (both quantitative and qualitative) approaches to investigate how the quality of life indices and scores can be interpreted within the same sample. Moreover, specific dry mouth clinical measures can be developed to include environmental and individual characteristics, in order to create direct, tailored and appropriate clinical interventions.

## Author Contributions

Both authors listed have made a substantial, direct and intellectual contribution to the work, and approved it for publication.

## Funding

The funds received for open access publication fees are from the University of Otago.

## Conflict of Interest

The authors declare that the research was conducted in the absence of any commercial or financial relationships that could be construed as a potential conflict of interest.

## Publisher's Note

All claims expressed in this article are solely those of the authors and do not necessarily represent those of their affiliated organizations, or those of the publisher, the editors and the reviewers. Any product that may be evaluated in this article, or claim that may be made by its manufacturer, is not guaranteed or endorsed by the publisher.

## References

[1] Fox RI. Sjögren's syndrome. Lancet. (2005) 366:321–31. 10.1016/S0140-6736(05)66990-5 16039337

[2] Al-Hashimi I. Xerostomia secondary to Sjögren's syndrome in the elderly. Drugs Aging. (2005) 22:887–99. 10.2165/00002512-200522110-00001 16323968

[3] Vitali C Bombardieri S Jonsson R Moutsopoulos H Alexander E Carsons S. Classification criteria for Sjögren's syndrome: a revised version of the European criteria proposed by the American-European Consensus Group. Ann Rheum Dis. (2002) 61:554–8. 10.1136/ard.61.6.554 12006334 PMC1754137

[4] Rasmussen A Ice JA Li H Grundahl K Kelly JA Radfar L. Comparison of the American-European Consensus Group Sjögren's syndrome classification criteria to newly proposed American College of Rheumatology criteria in a large, carefully characterised sicca cohort. Ann Rheum Dis. (2014) 73:31–8. 10.1136/annrheumdis-2013-203845 23968620 PMC3855629

[5] Shiboski CH Shiboski SC Seror R Criswell LA Labetoulle M Lietman TM. 2016 American College of Rheumatology/European League Against Rheumatism classification criteria for primary Sjögren's syndrome: a consensus and data-driven methodology involving three international patient cohorts. Ann Rheum Dis. (2017) 76:9–16. 10.1136/annrheumdis-2016-210571 27789466

[6] Binard A Devauchelle-Pensec V Fautrel B Jousse S Youinou P Saraux A. Epidemiology of Sjögren's syndrome: where are we now? Clin Exp Rheumatol. (2007) 25:1–4. 17417982

[7] Thomson WM. Issues in the epidemiological investigation of dry mouth. Gerodontology. (2005) 22:65–76. 10.1111/j.1741-2358.2005.00058.x 15934347

[8] Nederfors T Isaksson R Mörnstad H Dahlöf C. Prevalence of perceived symptoms of dry mouth in an adult Swedish population-relation to age, sex and pharmacotherapy. Commun Dent Oral Epidemiol. (1997) 25:211–6. 10.1111/j.1600-0528.1997.tb00928.x 9192149

[9] Shiboski CH Baer AN Shiboski SC Lam M Challacombe S Lanfranchi HE. Natural HIstory and predictors of progression to Sjögren's syndrome among participants of the Sjögren's international collaborative clinical alliance registry. Arthritis Care Res. (2018) 70:284–94. 10.1002/acr.23264 28437595 PMC5654699

[10] Ngo DYJ Thomson WM Nolan A Ferguson S. The lived experience of Sjögren's Syndrome. BMC Oral Health. (2016) 16:7. 10.1186/s12903-016-0165-4 26831141 PMC4736702

[11] Rojas-Alcayaga G Herrera Ronda A Espinoza Santander I Bustos Reydet C Rios Erazo M Wurmann P. Illness experiences in women with oral dryness as a result of Sjögren's syndrome: the patient point of view. Musculoskeletal Care. (2016) 14:233–42. 10.1002/msc.1134 27061842

[12] Enger TB Palm Ø Garen T Sandvik L Jensen JL. Oral distress in primary Sjögren's syndrome: implications for health-related quality of life. Eur J Oral Sci. (2011) 119:474–80. 10.1111/j.1600-0722.2011.00891.x 22112034

[13] Lackner A Ficjan A Stradner MH Hermann J Unger J Stamm T. It's more than dryness and fatigue: the patient perspective on health-related quality of life in Primary Sjögren's Syndrome-a qualitative study. PLoS ONE. (2017) 12:e0172056. 10.1371/journal.pone.0172056 28182787 PMC5300216

[14] Ware JE Jr Sherbourne CD. The MOS 36-item short-form health survey (SF-36): I. Conceptual framework and item selection. Med Care. (1992):473–83. 10.1097/00005650-199206000-00002 1593914

[15] Slade GD Spencer AJ. Development and evaluation of the oral health impact profile. Community Dent Health. (1994) 11:3–11. 8193981

[16] Atchison KA Dolan TA. Development of the geriatric oral health assessment index. J Dent Educ. (1990) 54:680–7. 10.1002/j.0022-0337.1990.54.11.tb02481.x 2229624

[17] Gibson B Periyakaruppiah K Thornhill MH Baker SR Robinson PG. Measuring the symptomatic, physical, emotional and social impacts of dry mouth: a qualitative study. Gerodontology. (2020) 37:132–42. 10.1111/ger.12433 31347735

[18] Fox PC Busch KA Baum BJ. Subjective reports of xerostomia and objective measures of salivary gland performance. J Am Dent Assoc. (1939) (1987) 115:581–4. 10.1016/S0002-8177(87)54012-0 3477595

[19] Navazesh M. Methods for collecting saliva. Ann N Y Acad Sci. (1993) 694:72–7. 10.1111/j.1749-6632.1993.tb18343.x 8215087

[20] Locker D. Xerostomia in older adults: a longitudinal study. Gerodontology. (1995) 12:18–25. 10.1111/j.1741-2358.1995.tb00125.x 8626175

[21] Al-Hashimi I Khuder S Haghighat N Zipp M. Frequency and predictive value of the clinical manifestations in Sjögren's syndrome. J Oral Pathol Med. (2001) 30:1–6. 10.1034/j.1600-0714.2001.300101.x 11140894

[22] Li Z Fu T Li L Cui Y Dong C Li J. Prevalence, severity, and predictors of dry eye and dry mouth in Chinese patients with primary Sjögren syndrome. Clin Rheumatol. (2018) 37:2971–9. 10.1007/s10067-018-4233-9 30094749

[23] Sisto M Lisi S Lofrumento D D'AMORE M Scagliusi P Mitolo V. Autoantibodies from Sjögren's syndrome trigger apoptosis in salivary gland cell line. Ann N Y Acad Sci. (2007) 1108:418–25. 10.1196/annals.1422.044 17894006

[24] Suresh L Malyavantham K Shen L Ambrus JL. Investigation of novel autoantibodies in Sjogren's syndrome utilizing Sera from the Sjogren's international collaborative clinical alliance cohort. BMC Ophthalmol. (2015) 15:38. 10.1186/s12886-015-0023-1 25881294 PMC4397858

[25] Martín-Nares E Hernández-Molina G. Novel autoantibodies in Sjögren's syndrome: a comprehensive review. Autoimmun Rev. (2019) 18:192–8. 10.1016/j.autrev.2018.09.003 30572138

[26] Li J Ha Y-M Kü N-Y Choi S-Y Lee SJ Oh SB. Inhibitory effects of autoantibodies on the muscarinic receptors in Sjögren's syndrome. Lab Investig. (2004) 84:1430–8. 10.1038/labinvest.3700173 15448705

[27] Konttinen YT Kasna-Ronkainen L. Sjögren's syndrome: viewpoint on pathogenesis. Scand J Rheumatol. (2002) 31:15–27. 10.1080/030097402317474883 12109538

[28] Alliende C Kwon Y-J Brito M Molina C Aguilera S Pérez P. Reduced sulfation of muc5b is linked to xerostomia in patients with Sjögren syndrome. Ann Rheum Dis. (2008) 67:1480–7. 10.1136/ard.2007.078246 17998215

[29] Humphrey SP Williamson RT. A review of saliva: normal composition, flow, and function. J Prosthet Dent. (2001) 85:162–9. 10.1067/mpr.2001.113778 11208206

[30] Soto-Rojas AE Kraus A. The oral side of Sjögren syndrome. Diagnosis and treatment. A review. Arch Med Res. (2002) 33:95–106. 10.1016/S0188-4409(01)00371-X 11886706

[31] Mathews SA Kurien BT Scofield RH. Oral manifestations of Sjögren's syndrome. J Dent Res. (2008) 87:308–18. 10.1177/154405910808700411 18362310

[32] Napeñas JJ Brennan MT Fox PC. Diagnosis and treatment of xerostomia (dry mouth). Odontology. (2009) 97:76–83. 10.1007/s10266-008-0099-7 19639449

[33] Guggenheimer J Moore PA. Xerostomia: etiology, recognition and treatment. J Am Dent Assoc. (2003) 134:61–9. 10.14219/jada.archive.2003.0018 12555958

[34] Berzon R Hays R Shumaker S. International use, application and performance of health-related quality of life instruments. Qual Life Res. (1993) 2:367–8. 10.1007/BF00422214 8161974

[35] Locker D. Measuring oral health: a conceptual framework. Commun Dent Health. (1988) 5:3–18. 3285972

[36] Brazier JE Harper R Jones N O'cathain A Thomas K Usherwood T. Validating the SF-36 health survey questionnaire: new outcome measure for primary care. Br Med J. (1992) 305:160–4. 10.1136/bmj.305.6846.160 1285753 PMC1883187

[37] Fernandes MJ Ruta DA Ogden GR Pitts NB Ogston SA. Assessing oral health-related quality of life in general dental practice in Scotland: validation of the OHIP-14. Commun Dent Oral Epidemiol. (2006) 34:53–62. 10.1111/j.1600-0528.2006.00254.x 16423032

[38] Segal B Bowman SJ Fox PC Vivino FB Murukutla N Brodscholl J. Primary Sjögren's Syndrome: health experiences and predictors of health quality among patients in the United States. Health Qual Life Outcomes. (2009) 7:1–9. 10.1186/1477-7525-7-46 19473510 PMC2693523

[39] Cui Y Li L Xia L Zhao Q Chen S Fu T. The impact of disease activity and psychological status on quality of life for Chinese patients with primary Sjögren's syndrome. Patient Preference Adherence. (2018) 12:1513. 10.2147/PPA.S163417 30174416 PMC6110269

[40] Meijer JM Meiners PM Huddleston Slater JJ Spijkervet FK Kallenberg CG Vissink A. Health-related quality of life, employment and disability in patients with Sjögren's syndrome. Rheumatology. (2009) 48:1077–82. 10.1093/rheumatology/kep141 19553376

[41] Stewart CM Berg KM Cha S Reeves WH. Salivary dysfunction and quality of life in Sjögren syndrome: a critical oral-systemic connection. J Am Dent Assoc. (2008) 139:291–9. 10.14219/jada.archive.2008.0158 18310733

[42] Rusthen S Young A Herlofson BB Aqrawi LA Rykke M Hove LH. Oral disorders, saliva secretion, and oral health-related quality of life in patients with primary Sjögren's syndrome. Eur J Oral Sci. (2017) 125:265–71. 10.1111/eos.12358 28643390

[43] Al Hamad A Lodi G Porter S Fedele S Mercadante V. Interventions for dry mouth and hyposalivation in Sjögren's syndrome: a systematic review and meta-analysis. Oral Dis. (2019) 25:1027–47. 10.1111/odi.12952 30086205

[44] Valtýsdóttir ST Gudbjörnsson B Lindqvist U Hällgren R Hetta J. Anxiety and depression in patients with primary Sjögren's syndrome. J Rheumatol. (2000) 27:165–9. 10648034

[45] Vivino FB. Sjogren's syndrome: clinical aspects. Clin Immunol. (2017) 182:48–54. 10.1016/j.clim.2017.04.005 28428095

[46] Gandía M Morales-Espinoza EM Martín-González RM Retamozo MS Kostov B Belenguer-Prieto R. Factors influencing dry mouth in patients with primary Sjögren syndrome: usefulness of the ESSPRI index. Oral Health Dent Manag. (2014) 13, 402–7. 24984655

[47] Ferrans CE Zerwic JJ Wilbur JE Larson JL. Conceptual model of health-related quality of life. J Nurs Scholarsh. (2005) 37:336–42. 10.1111/j.1547-5069.2005.00058.x 16396406

[48] Masood M Masood Y Newton TJ. Methods of qualitative research in dentistry: a review. Dental Update. (2010) 37:326–36. 10.12968/denu.2010.37.5.326 20669712

[49] Wilson IB Cleary PD. Linking clinical variables with health-related quality of life: a conceptual model of patient outcomes. JAMA. (1995) 273:59–65. 10.1001/jama.273.1.59 7996652

[50] Engel GL. The clinical application of the biopsychosocial model. J Med Philos. (1981) 6:101–23. 10.1093/jmp/6.2.101 7264472

